# Supplementary material for: Spatial Variation in Stream-Water Bacterial Communities Across Sites Differing in Wild Chinese Giant Salamander Detection Frequency
Source: Biology (Basel). 2026 Sep 18;15(18):1647. doi: 10.3390/biology15181647 (PMC13604818; doi:10.3390/biology15181647)
Supplement: Supplementary file 1 [file biology-15-01647-s001.zip › Data Related to “Spatial Variation in Stream-Water Bacterial Communities across Sites Differing in Wild Chinese Giant Salamander Detection Frequency”/Microbial Data/report/src/hyperlinks/QC_methods_LRS_CH.pdf]

中文版 Method

仅供客户在文章写作时参考，分析内容和方法请以结题报告

为准， 请客户自行承担文章查重等相关风险。

1 实验流程

1.1 DNA 检测

根据样本类型，进行DNA提取，将提取后的 DNA 通过 1%琼脂糖凝胶电泳（120V，30min）进行质量检测，评估其条带完整性和核酸纯度。将符合要求的 DNA 样本用无菌无核酸酶水稀释至终浓度 1ng/μL，用于后续实验。

1.2 PCR 产物的获取

以稀释后的基因组 DNA 为模板，采用带有特异性条形码（barcode）的引物对目标 16S rRNA 基因区域进行 PCR 扩增。所 有 PCR 混 合 液 加 入 12.5 μL KAPAHiFi HotStart ReadyMix、2.5 μM 引物和10 ng基因组DNA模板， 在95℃下进行3分钟的第一次变性，然后在98℃ (20s)、57℃ (30s) 和 72℃ (75s) 下进行22次循环， 最后在72℃下保持5分钟。扩增产物经 2%琼脂糖凝胶电泳验证后，使用磁珠纯化试剂（如 AMPure XP）去除引物二聚体。

1.3 Kinnex 文库构建

将不同样本的barcode 标记 PCR 产物等摩尔混合，采用 Kinnex 长读长测序技术进行文库构建，具体步骤如下：

末端修复：使用 Kinnex 末端修复酶（20℃，30min）处理 PCR 产物，确保DNA 末端平整化。

接头连接：加入 Kinnex 特异性测序接头（20μM）及 T4 DNA 连接酶（20℃，15min），使 DNA 片段与测序适配体连接。

线性阵列组装：通过 Kinnex 组装酶（37℃，1h）将 16S rRNA 基因片段串联成长片段，形成线性 DNA 阵列。

文库纯化：采用SMRTbell®磁珠进行片段筛选（0.45×和 0.8×磁珠比例），保留 500–2000bp 的目标片段，以提高文库质量。

1.4 文库质量评估与测序

纯化后的文库使用Qubit® 4.0 荧光定量仪测定浓度，并通过 Agilent 2100 检测片段大小分布，确保主峰位于预期范围（1.5–3kb）。符合质量标准的文库在 Revio 测序平台上进行单分子实时（SMRT）测序，以获得高质量的长读长测序数据。
